# Factors predicting biochemical response and survival benefits following radioligand therapy with [^177^Lu]Lu-PSMA in metastatic castrate-resistant prostate cancer: a review

**DOI:** 10.1007/s00259-021-05237-y

**Published:** 2021-03-06

**Authors:** Reyhaneh Manafi-Farid, Sara Harsini, Bahare Saidi, Hojat Ahmadzadehfar, Ken Herrmann, Alberto Briganti, Jochen Walz, Mohsen Beheshti

**Affiliations:** 1 Research Center for Nuclear Medicine, Tehran University of Medical sciences, Tehran, Iran; 2 Association of Nuclear Medicine and Molecular Imaging (ANMMI), Universal Scientific Education and Research Network (USERN), Tehran, Iran; 3 Department of Nuclear Medicine, Klinikum Westfalen, Dortmund, Germany; 4 Department of Nuclear Medicine, University Hospital, Essen, Germany; 5 Urological Research Institute, Scientific Institute San Raffaele, Vita-Salute San Raffaele University, Milan, Italy; 6 Department of Urology, Institute Paoli-Calmettes Cancer Centre, Marseille, France; 7 Division of Molecular Imaging and Theranostics, Department of Nuclear Medicine & Endocrinology, Paracelsus Medical University, Salzburg, Austria; 8 Department of Nuclear Medicine, University Hospital, RWTH University, Aachen, Germany

**Keywords:** [^177^Lu]Lu-PSMA, Radioligand therapy, Predictive factors, Response to therapy, Prognosis

## Abstract

**Background:**

Prostate cancer (PC) is one of the most common cancers in men. Although the overall prognosis is favorable, the management of metastatic castration-resistant prostate cancer (mCRPC) patients is challenging. Usually, mCRPC patients with progressive disease are considered for radioligand therapy (RLT) after exhaustion of other standard treatments. The prostate-specific membrane antigen (PSMA) labeled with Lutetium-177 ([^177^Lu]Lu-PSMA) has been widely used, showing favorable and successful results in reducing prostate-specific antigen (PSA) levels, increasing quality of life, and decreasing pain, in a multitude of studies. Nevertheless, approximately thirty percent of patients do not respond to [^177^Lu]Lu-PSMA RLT. Here, we only reviewed and reported the evaluated factors and their impact on survival or biochemical response to treatment to have an overview of the potentialprognostic parameters in [^177^Lu]Lu-PSMA RLT.

**Methods:**

Studies were retrieved by searching MEDLINE/PubMed and GoogleScholar. The search keywords were as follows: {(“177Lu-PSMA”) AND (“radioligand”) AND (“prognosis”) OR (“predict”)}. Studies discussing one or more factors which may be prognostic or predictive of response to [^177^Lu]Lu-PSMA RLT, that is PSA response and survival parameters, were included.

**Results:**

Several demographic, histological, biochemical, and imaging factors have been assessed as predictive parameters for the response to thistreatment; however, the evaluated factors were diverse, and the results mostly were divergent, except for the PSA level reduction after treatment, which unanimously predicted prolonged survival.

**Conclusion:**

Several studies have investigated a multitude of factors to detect those predicting response to [^177^Lu]Lu-PSMA RLT. The results wereinconsistent regarding some factors, and some were evaluated in only a few studies. Future prospective randomized trials are required to detect theindependent prognostic factors, and to further determine the clinical and survival benefits of [^177^Lu]Lu-PSMA RLT.

## Introduction

Prostate cancer (PC) is one of the most common cancers in men [1]. Although the prognosis is generally favorable [2], the treatment of PC is challenging in the cases of metastatic castration-resistant prostate cancer (mCRPC) [3]. The prostate-specific membrane antigen (PSMA) is a transmembrane protein and highly expressed in PC cells [1, 4]. Radioligands targeting PSMA are promising agents for the imaging and treatment of PC patients [1, 5–7].

Different ligands of PSMA have been developed and labeled with various radioisotopes for imaging and therapeutic purposes [8–16]. PSMA-617 labeled with Lutetium-177 ([^177^Lu]Lu) [17] is more commonly used in the clinics for the treatment of PC. Additionally, alpha emitters, such as Actinium-225 and Bismuth-213 [18, 19], have been labeled with PSMA and employed for treatment of mCRPC.

Although the phase III clinical trial is still ongoing, [^177^Lu]Lu-PSMA RLT is widely accepted in countries where it is available and seems to be well-tolerated. It has been successfully employed reducing prostate-specific antigen (PSA) levels, increasing the quality of life, decreasing pain and analgesic intake, in a multitude of studies [4, 20–27]. [^177^Lu]Lu-PSMA RLT seems safe even in patients at advanced stages of the disease [28]. A phase II trial demonstrated that 57% of patients achieve a biochemical response, described as ≥50% PSA reduction 12 weeks after therapy [21]. It should be emphasized that the response rate is reportedly variable [29]. The OS ranges between 7.5 and 15 months and progression-free survival (PFS) between 4.5 and 13.7 months [29].

Since approximately 30% of patients do not respond to this expensive treatment [22], it is still of concern to predict the outcome and individualize the treatment considering its potential benefits. Furthermore, [^177^Lu]Lu-PSMA RLT is administered in almost all of the reviewed studies as a “last line” treatment. Most of the patients have already received chemotherapy, abiraterone, or enzalutamide, as well as [^233^Ra]Radium-dichloride ([^223^Ra]RaCl) or external radiation therapy, if they were not contraindicated. Hence, the moderate survival benefit in these heavily pre-treated patients would be of importance. So far, several demographic, histological, biochemical, and imaging factors have been assessed as predictive parameters for a response to [^177^Lu]Lu-PSMA RLT. Here, we reviewed the evaluated factors and their impact on survival or biochemical response to treatment to have an overview of the potential prognostic parameters in [^177^Lu]Lu-PSMA RLT.

## Methods

In this narrative review, studies were retrieved by searching the following literature databases in September 2020: MEDLINE/PubMed and Google Scholar. No language or time limitation was applied in all the process of searching. Moreover, the references of the included and relevant systematic review studies were searched manually. The search keywords were as follows: {(“177Lu-PSMA”) AND (“radioligand”) AND (“prognosis”) OR (“predict”)}. Studies discussing one or more factors which may be prognostic or predictive of response to [177Lu]Lu-PSMA RLT, that is, PSA response and survival parameters, were included. Since the 177Lu-PSMA is employed for the treatment of mCRPC patients less than a decade, we used the option of showing the results for every year, separately, to simplify the search.

Articles were excluded if they were a review, case report, letter, guideline, and articles on radiochemistry, preclinical studies, biodistribution, or dosimetry. Following retrieving the pertinent articles, two autonomous reviewers screened the title and abstract of all the included studies according to the inclusion criteria. In the next step, the full texts of included articles were reviewed in detail. Any disagreements were resolved by consensus. The following data were extracted from each included paper: title, first author, year of publication, sample size, age, initial PSA value, PSA doubling time, baseline PSA value, prior therapies, Gleason score, activity and number of [^177^Lu]Lu-PSMA-RLT cycles, PSA decline, the intensity of uptake on PET/CT, visceral metastasis, bone metastasis, lymph node metastasis, level of alkaline phosphatase, level of lactate dehydrogenase, bone marrow status, level of other serum markers, performance status, and analgesic intake (Table 1).

**Table 1:** The impact of different factors with a predictive potential for response to therapy or longer overall survival in response to [^177^Lu]Lu-PSMA RLT

Confirmed	Positive impact	Any PSA decline after the 1st cycle*
	Negative impact	- Gleason score
		- Lymph node metastasis
Plausible	Positive impact	- Any PSA decline after a few cycles*^†^
		- > 50% PSA decline after a few cycles*^†^
		- Better performance status
	Negative impact	- Visceral metastasis
		- Increased ALP
		- Higher CRP^‡^
		- Regular need for analgesic drugs^‡^
	No impact	- Initial PSA
		- PSA doubling time
		- Prior [^223^Ra]RaCl
		- Prior ARTA
		- Administered activity
		- WBC count
		- Plt count
Controversial	Positive impact	- > 50% PSA decline after the 1st cycle*
		- Higher intensity of uptake in the pre-therapy scan
		- Higher cumulative activity
		- Higher number of cycles
	Negative impact	- Prior chemotherapy
		- PSA at the time of [^177^Lu]Lu-PSMA RLT
		- Bone metastasis
		- Increased LDH
		- Lower Hb
		- Lower albumin
		- Higher LFT
		- Younger age

*[*^*177*^*Lu]Lu-PSMA RLT*, Lutetium-177 prostate-specific membrane antigen radioligand therapy; *[*^*223*^*Ra]RaCl*, Radium-223 dichloride therapy; *Alb*, albumin; *ALP*, alkaline phosphatase; *ARTA*, androgen receptor targeting agent therapy; *CRP*, C-reactive protein; *Hb*, hemoglobin; *LDH*, lactate dehydrogenase; *LFT*, liver function test; *Plt*, platelet; *PSA*, prostate-specific antigen; *WBC*, white blood cell

*Cycle of [^177^Lu]Lu-PSMA RLT

^†^Three cycles in the most studies

^‡^Evaluated in a few studies

## Predictive factors

### Initial PSA and PSA doubling time

The impact of initial PSA level and PSA doubling time in predicting [^177^Lu]Lu-PSMA RLT has rarely been discussed in the literature. Initial PSA level did not show any noticeable association with OS in the study performed by Rahbar et al., analyzing mCRPC patients treated with [^177^Lu]Lu-PSMA RLT [23]. In another investigation, Bräuer et al. assessed 59 mCRPC patients, who had been treated with at least one next-generation anti-hormonal drug as well as chemotherapy before [^177^Lu]Lu-PSMA RLT, and depicted that initial PSA level (cut-off value of 350 ng/mL) and pre-treatment PSA doubling time (cut-off < 3 months) were not associated with PFS or OS [30]. Also, the findings of the study performed by Ferdinandus et al. revealed that PSA doubling time prior to the administration of [^177^Lu]Lu-PSMA was deemed not prognostic for OS [31]. Therefore, the initial PSA level and its doubling time apparently have no significant impact on OS or PFS following [^177^Lu]Lu-PSMA RLT warranting further confirmation.

### PSA at the time of [^177^Lu]Lu-PSMA RLT

Hypothetically, the higher baseline PSA may suggest a higher tumoral burden and possibly worse outcome. Studies evaluating the impact of baseline PSA level on patients’ response to treatment or survival did not reach a consensus on this matter.

Yordanova et al. [32] outlined that baseline PSA level significantly correlates with survival following [^177^Lu]Lu-PSMA RLT, as patients with PSA values lower than 47 ng/mL at baseline achieved a longer OS than patients with higher PSA levels (20 vs. 11 months). In another study, Gafita et al. [33] delineated that baseline PSA level significantly associates with OS in the multivariable analysis (HR: 1.63, *P* = 0.007) but not with the imaging-based PFS. Also, a retrospective analysis by Barber et al. [34] signified that baseline PSA of greater than 60 ng/mL is a significant determinant of inferior OS in taxane-pre-treated, taxane-naïve, and entire cohort (HR: 2.59 [*P* = 0.008], HR:2.40 [*P* = 0.019], and HR: 3.15 [*P* < 0.001], respectively), as well as inferior radiographic PFS in the taxane-naïve subgroup (HR: 1.88 [*P* = 0.02]) and entire cohort (HR: 1.92 [*P* < 0.001]). However, none of these factors kept their statistical significance in the multivariable analysis [34]. Likewise, Heck et al. [20] notified baseline PSA level as the predictor of OS only in the univariate analysis of 100 mCRPC patients undergoing [^177^Lu]Lu-PSMA RLT (HR: 1, *P* = 0.007), but not of clinical PFS or PSA response.

On the contrary, PSA level measured 1 day before [^177^Lu]Lu-PSMA RLT did not show a negative impact on the response to [^177^Lu]Lu-PSMA RLT, in a study by Ferdinandus et al. [24]. The same results were reported by other authors who did not show any significant association between baseline PSA level and patients’ OS after [^177^Lu]Lu-PSMA RLT [31, 32, 35, 36]. Gafita et al. [37] found no association between PSA level and either PFS or OS, in a cohort with the median PSA baseline of 126 ng/ml (IQR: 37–368). Moreover, Derlin et al. [38] found no significant association between the PSA level at day 1 of cycle 1 and PSA change following [^177^Lu]Lu-PSMA RLT. Additionally, baseline PSA level was found not to be associated with a PSA decline of ≥ 20% following [^177^Lu]Lu-PSMA RLT [39] or any PSA response [40].

In summary, the prognostic value of baseline PSA level has been controversially discussed, which warrants further investigations.

### PSA decline

The decrease in PSA level after treatment is used as a surrogate marker for response in prostate cancer. Reviewing the literature, most authors have reported PSA decline ≥ 50% or any PSA decline 2 weeks to 3 months after the first or last cycle of [^177^Lu]Lu-PSMA RLT, based on Prostate Cancer Clinical Trial Working Group criteria [21, 41, 42], and have correlated the data with survival and potentially predictive factors [4, 20, 21, 30].

Several studies indicated a significant correlation between longer survival and any PSA decline after the first cycle of [^177^Lu]Lu-PSMA RLT [17, 23, 30, 39]. Regarding ≥ 50% PSA decline after the first cycle, some studies did not find a predictive value of ≥ 50% PSA decline for longer OS [17, 23, 30, 33]. Additionally, there was no difference in OS between patients achieving < 50% or ≥ 50% decrease in PSA level [23, 30] with Ahmadzadehfar et al. [4] reporting that the median OS is the same for patients with < 50% and ≥ 50% decrease in PSA level, measured 8 weeks after the first cycle (13.9 vs. 14.3 months, respectively). On the other hand, Gadot et al. [39] showed that ≥ 50% and ≥ 20% PSA decline after the first cycle correlates with longer OS (11 vs. 3.6 months). Also, Yadav et al. [26] depicted that ≥ 50% PSA decline predicts better OS compared with < 50% decline (13 vs. 16 months), only in the univariate analysis [26]. Moreover, Gafita et al. [33] showed that ≥ 30% PSA decline after 6 weeks of therapy correlates with imaging-based PFS.

Finally, in a meta-analysis, Kim and Kim [22] correlated the biochemical response after the first cycle of [^177^Lu]Lu-PSMA RLT with OS. They found that any PSA decline occurs in 68% (95% confidence interval [CI], 63–72%], and ≥ 50% PSA decline in 34% (95% CI, 30–38%) [22]. The pooled hazard ratio (HR) for OS was 0.29 (95% CI, 0.21–0.40 *P* < 0.00001) for any PSA decline. Nevertheless, ≥ 50% PSA decline was not predictive of OS (HR: 0.82 [95% CI, 0.54–1.25]; *P* = 0.39) [22]. However, there were limitations to this meta-analysis. All of the included studies were retrospective, and the patients had been received different therapeutic regimens before [^177^Lu]Lu-PSMA RLT. Also, some studies had a small sample size.

Aside from PSA decline after the first cycle, in another meta-analysis, Yadav et al. [43] evaluated PSA response after variable numbers of cycles of [^177^Lu]Lu-PSMA RLT. Any PSA decline was seen in 75% (95% CI, 70–79%) and ≥ 50% PSA decline in 46% (95% CI, 40–53%), reportedly [43]. They did not correlate the PSA response with OS since the results were heterogeneous [43]. Considering the prognostic value, after full cycle treatment or a few cycles of therapy, any PSA decline after [^177^Lu]Lu-PSMA RLT (mostly 3 cycles) predicted longer OS [17, 26], approximately 17.5 vs. 8 months [17]. Furthermore, PSA decline ≥ 50% predicted longer PFS and OS (after median of 3 cycles), in multiple studies [20, 21]. The PSA PFS was 9.9 months in responders versus 4.1 months in non-responders [21]. Also, Ahmadzadehfar et al. [17] showed that patients with PSA decline ≥ 50% after the third cycle compared to baseline PSA have longer OS (approximately 17 vs. 10 months). Additionally, in a prospective study by Yadav et al. [26], PSA decline ≥ 50% predicted longer OS in the multivariate analysis (HR: 8.07 [95% CI, 0.2607–0.7786]).

Recently, a repeated course of [^177^Lu]Lu-PSMA RLT is being administered to some patients with a prior excellent response [44, 45] and authors are investigating the prognostic factors [29]. They showed that rechallenge therapy is safe. Although patients with ≥ 50% PSA decline after the first cycle lived longer than those with an increase in PSA level, the difference was not statistically significant [29].

Although it may suggest that most of the patients showing PSA decline after the first cycle of therapy would have a better outcome, some studies reported that 30–50% of the patients with no PSA decline after the first cycle show the decrease in PSA level after second or third cycles [17, 46]. On the other hand, the possibility of the flare phenomenon has been discussed after [^177^Lu]Lu-PSMA RLT and the assumption that PSA increase may not always indicate disease progression [46]. However, Gafita et al. [33] recently demonstrated that the PSA flare is very uncommon after [^177^Lu]Lu-PSMA RLT. Interestingly, while the decrease in PSA level predicted longer survival, the increase in PSA levels did not associate with worse outcome [20, 26].

In summary, PSA decline after [^177^Lu]Lu-PSMA RLT is a valuable factor to predict the outcome. Any PSA decline after the first treatment cycle is the most definite prognosticator of longer OS. Also, ≥ 50% PSA decline after the treatment conclusion appears as an important predictor of survival. The results regarding ≥ 50% PSA decline after the first cycle are controversial. In addition, it is of great importance to determine unified treatment protocols and endpoints of evaluating the therapy response. It should be also noted that although the PSA level is used for evaluation of the disease progression or response to therapy, there are patients having the radiologically progressive disease (e.g., osteoblastic bone metastases) without changes in PSA level. Thus, functional imaging (e.g., PET/CT) may play an important role, particularly in treatment assessment of such cases.

### Prior therapies

[^177^Lu]Lu-PSMA RLT is a novel therapeutic approach showing promising results. However, most of the patients receive [^177^Lu]Lu-PSMA RLT after the failure of other standard treatments, including conventional and new anti-hormonal drugs, chemotherapy, or [^223^Ra]RaCl [47]. The impact of such prior therapies on survival or PSA response has been addressed in multiple studies showing controversial results.

In different studies, prior treatments, including anti-hormonal drugs, chemotherapy, or [^223^Ra]RaCl, in advanced mCRPC patients did not correlate with PSA response, clinical PFS, or OS [20, 24, 28, 40]. In detail, the results of the study performed by Rahbar et al. [23] did not show second-line chemotherapy or prior [^223^Ra]RaCl to affect OS in the heavily pre-treated advanced mCRPC patients. Bräuer et al. [30], in the same manner, depicted a lack of association between prior chemotherapy and OS following administration of [^177^Lu]Lu-PSMA. Likewise, Ahmadzadehfar et al. [35] studied the influence of the history of chemotherapy on OS, in 100 mCRPC patients who had already received either abiraterone or enzalutamide prior to [^177^Lu]Lu-PSMA RLT. They concluded that there is no significant difference in median OS between patients with the history of chemotherapy (approximately 14 months) and chemotherapy-naïve patients (approximately 15.5 months). Barber et al. [34] revealed an association between prior taxane chemotherapy and inferior OS and radiographic PFS in the univariate analysis (HR: 2.55 [*P* < 0.001], and HR: 1.71 [*P* = 0.003], respectively), none of which remained independent in the multivariable analysis. It should be kept in mind that this study was retrospective, and the patients that received chemotherapy were at higher risk of disease progression and had more adverse prognostic features. Hence, shorter OS would be expected for taxane-pre-treated patients, as it is evident in the multivariable analysis that factors other than the history of chemotherapy may influence the survival. This selection bias is plausible for all other studies with the retrospective nature.

Furthermore, according to the simple linear regression analysis by Derlin et al. [38], it has been demonstrated that previous treatments, including abiraterone, enzalutamide, chemotherapy, and external radiation therapy, do not have a significant association with change in PSA following [^177^Lu]Lu-PSMA RLT. The use of abiraterone, steroid, or previous radiotherapy, as depicted by Suman et al. [48], did not stand as predictors of PFS or OS following [^177^Lu]Lu-PSMA RLT. Also, the history of using abiraterone or enzalutamide or chemotherapy before [^177^Lu]Lu-PSMA was not predictive of either any or ≥ 50% PSA decline following [^177^Lu]Lu-PSMA RLT [26].

There are, on the other hand, some reports delineating a significant association between the existence or the number of received treatments with PSA response or OS following [^177^Lu]Lu-PSMA RLT. Ahmadzadehfar et al. [4] analyzed 416 patients who had received both abiraterone and enzalutamide (53.6%), chemotherapy with docetaxel (75.5%), chemotherapy with cabazitaxel (26.4%), and [^223^Ra]RaCl (20.4%) before [^177^Lu]Lu-PSMA RLT. They suggested prior chemotherapy as a significant prognosticator of shorter OS in both univariate and multivariate analyses [4]. The median OS in patients who had received one or two lines of chemotherapy with docetaxel or docetaxel followed by cabazitaxel was 10.9 and 8.9 months, respectively, which was significantly shorter than in patients without any prior chemotherapy (median OS of 14.6 months) [4]; however, no difference was noted in OS between patients who avoided to receive chemotherapy and patients for whom chemotherapy was contraindicated [4]. Other prior therapies, including anti-hormonal therapy and [^223^Ra]RaCl, did not reveal any significant impact on OS [4]. As stated in Gafita et al.’s multivariate analysis [33], positive chemotherapy status prior to [^177^Lu]Lu-PSMA RLT was significantly associated with imaging-based PFS (HR = 1.75, *P* = 0.04), but not with OS.

Gadot et al. [39] demonstrated that neither the number of total prior treatment lines for CRPC nor prior treatment with [^223^Ra]RaCl was associated with PSA decline of more than 20%; nevertheless, there was a negative association between the number of previous chemotherapy lines (ranging from 0 to 2) and a PSA decline above 20% (*P* = 0.043).

Finally, Kulkarni et al. [49] categorized 224 mPC patients according to prior therapies, including chemotherapy (*n* = 110, second-line with cabazitaxel *n* = 20), androgen deprivation therapy (*n* = 206), newer anti-cancer agents (abiraterone [*n* = 91] and enzalutamide [*n* = 79]), [^223^Ra]RaCl (*n* = 19), and no previous therapy (*n* = 18). They showed that the first-line [^177^Lu]Lu-PSMA RLT was associated with the longest OS (median not reached at 55 months, since all patients were alive) [49]. The previous chemotherapy correlated with significantly shorter survival in comparison with chemotherapy-naïve patients (19 vs. 38 months) [49]. Moreover, patients with the history of previous [^223^Ra]RaCl had shorter OS, and those with the addition of abiraterone or enzalutamide had significantly prolonged survival [49]. Hence, previous first- or second-line chemotherapy and [^223^Ra]RaCl were proposed as predictors of worse survival. In contrast, the addition of newer androgen receptor–targeted agents was suggested to have a synergistic effect in combination with [^177^Lu]Lu-PSMA RLT [49].

Overall, it seems that except for chemotherapy, other prior therapies have no impact on outcome following [^177^Lu]Lu-PSMA RLT. However, patients receiving chemotherapy usually have unfavorable characteristics, which might cause bias in the results of the published studies. Further researches and systematic reviews concerning each therapy, with particular attention to chemotherapy, are required to draw a definite conclusion. Also, early administration of [^177^Lu]Lu-PSMA RLT is another interesting subject for future trails.

### Activity and number of [^177^Lu]Lu-PSMA-RLT cycles

A standard administered activity of [^177^Lu]Lu-PSMA has not been established by prospective trials, thus far, a wide range of activities (up to 9.3 GBq) has been used in safety and toxicity trials. The conflicting results regarding the impact of the number of [^177^Lu]Lu-PSMA RLT cycles or the administered cumulative activity have been documented on patients’ response to treatment or survival following [^177^Lu]Lu-PSMA RLT.

Rahbar et al. [23] indicated that the cumulative injected activity of ≥ 18.8 GBq is a prognosticator of a longer OS in a total of 104 patients treated with 351 cycles of [^177^Lu]Lu-PSMA RLT (median OS of 14.5 vs. 12.0 months in those receiving cumulative activity of ≥ 18.8 and < 18.8 GBq, respectively, HR: 0.53). However, as patients with longer survival had a higher chance of receiving higher cumulative activity, the inhomogeneous number of cycles in this study should be kept in mind. Furthermore, according to the multivariate analysis of 167 mCRPC who underwent [^177^Lu]Lu-PSMA RLT, the cumulative administered activity of more than 16 GBq was found to be associated with inferior OS in both the taxane-pre-treated subgroup (HR = 0.37, *P* = 0.002) and the entire cohort (HR = 0.5, *P* = 0.005), but not in the taxane-naïve subgroup [34]. On the other hand, Ferdinandus et al. [24] evaluated 40 patients receiving one cycle of [^177^Lu]Lu-PSMA with a mean dose of 6.0 GBq (range: 4.1–7.1 GBq) and a mean activity of 78.5 MBq/kg of body weight and demonstrated that PSA response was independent of these factors. Also, in other studies, the cumulative activity was not predictive of PSA response [28, 50] or OS [48].

In another study of 145 mCRPC patients treated with 1–4 cycles (activity range: 2–8 GBq per cycle), Rahbar et al. [28] demonstrated that patients with a higher number of therapy cycles (≥ 3) had a higher rate of PSA response (odds ratio: 5.83, *P* = 0.02) in contrast to per cycle administered activity. The number of therapy cycles remained associated with the PSA response rate in the multivariate analysis (*P* ≤ 0.05) [28]. Likewise, Kesavan et al. [50] studied 20 progressive mCRPC patients with a mean prescribed activity of 5.5 GBq per patient. They showed that patients receiving three cycles of therapy were statistically more likely to experience ≥ 50% reduction in PSA compared to those treated with one, two, or four cycles (*P* < 0.0001) [50].

Additionally, Rathke et al. [51], although in a small patient group of 40 patients, demonstrated that four different treatment activities of [^177^Lu]Lu-PSMA (4, 6, 7.4, and 9.3 GBq) did not influence the PSA response.

Moreover, Yordanova et al. studied [^177^Lu]Lu-PSMA RLT rechallenge in 30 patients after a median of 6 months (range 2–26) with a median of 3 (range 1–6) rechallenge cycles and showed that the median OS was significantly longer in patients after rechallenge [^177^Lu]Lu-PSMA RLT compared to those received only baseline therapy (12 vs. 9 months, *P* < 0.001) [29].

Overall, the studies concerning the impact of the administered activity, cumulative dose, and number of cycles on outcome following [^177^Lu]Lu-PSMA RLT are inadequate, and results are controversial. Further trials would clarify the influence of these factors to help determine a standard protocol.

### Intensity of uptake in positron emission tomography/computed tomography

The intensity of uptake in [^68^Ga]Ga-PSMA PET/CT represents the PSMA expression in prostate tumoral cells. Pre-treatment imaging is used before [^177^Lu]Lu-PSMA RLT to document the presence of PSMA-avid lesions. Although the exact amount of PSMA avidity required for treatment has not been established yet, some centers consider 1.5 as the least ratio of the mean standardized maximum value (SUVmean) of the lesion-to-liver [21]. It is hypothesized that the higher [^68^Ga]Ga-PSMA uptake may correlate with higher [^177^Lu]Lu-PSMA uptake and better response to treatment. However, the evaluated parameters are heterogeneous and results are inconsistent, in the literature.

The absorbed doses of [^177^Lu]Lu-PSMA RLT positively correlated with mean whole-body uptake on [^68^Ga]Ga-PSMA PET/CT in a study by Violet et al., reporting unfavorable PSA response in patients with lower absorbed doses [52]. Seifert et al. [53] showed that average maximum SUV (SUVmax) of the lesions on [^68^Ga]Ga-PSMA PET/CT was a significant prognosticator of OS in contrast to the maximum and minimum SUVmax values. Patients with lower average SUVmax of the lesions and low PSMA expressing metastases had shorter OS (5.3 vs. 15.1 months and 7.9 vs. 21.3 months, respectively) [53]. Also, the change in SUVmax of the metastatic lesions may have an association with PSA response [54].

Emmett et al. [55] correlated the response to [^177^Lu]Lu-PSMA RLT with pre-treatment 2-[^18^F]FDG and [^68^Ga]Ga-PSMA PET/CT images in a prospective study with 14 patients. No imaging parameter predicted ≥ 50% PSA reduction [55]. However, higher pre-treatment SUVmax and SUVmean on [^68^Ga]Ga-PSMA PET/CT were predictive of ≥ 30% PSA reduction (SUVmax: 17 ± 9 versus 44 ± 15, *P* < 0.007; SUVmean: 6 ± 4 versus 10 ± 4, *P* < 0.04) [55]. Interestingly, they reported that none of the patients with SUVmax less than 15 on [^68^Ga]Ga-PSMA PET/CT had a biochemical response to [^177^Lu]Lu-PSMA RLT [55]. Also, Seifert et al. [53] reported the best cut-off value of 14.3 for average SUVmax of the lesions as the predictor of OS [53].

Heinzel et al. [56] correlated the decrease in SUVmean (≥ 30%) on [^68^Ga]Ga-PSMA PET/CT, after at least 3 cycles of therapy, with PSA response (≥ 50% decline). The fitted receiver operating characteristic area was 0.70, and the difference in OS was not statistically significant between responders and non-responders (19.6 vs. 15.9, respectively) [56]. Also, the only [^68^Ga]Ga-PSMA PET/CT-predictive parameter of longer OS was higher whole-body SUVmean (9.8 vs. 6.3 months) [31]. Additionally, higher pre-treatment total tumor volume (HR: 0.87) and SUVmean (HR: 0.94) predicted longer PSA PFS in a study by Gafita et al. [37]. However, Grubmüller et al. [36] assessed the changes in total tumor volume (from the pre-treatment to the post-treatment scan) and showed significant associations with PSA response and OS in contrast to changes in SUVmean [36].

In another study, Ferdinandus et al. [24] revealed no correlation between SUVmax of the metastatic lesions in different organs and response to therapy. Yadav et al. [26] showed that pre-treatment peak standardized uptake value corrected for lean body mass for different sites of metastases does not predict PSA response.

Moreover, Yordanova et al. [29] employed post-treatment [^68^Ga]Ga-PSMA PET/CT to predict survival after rechallenge [^177^Lu]Lu-PSMA RLT and failed to prove any prognostic value.

The impact of the intensity of [^68^Ga]Ga-PSMA uptake is discussed controversially in the literature using inhomogeneous patient’s population with different imaging protocols and quantitative approaches. Nevertheless, most studies were not able to predict response using tracer intensity alone. Hence, making the decision for [^177^Lu]Lu-PSMA RLT should not be based on a predefined cut-off for [^68^Ga]Ga-PSMA uptake in pre-treatment PET/CT.

### Visceral metastasis

The prognostic value of the presence of visceral metastasis has been correlated with survival parameters or biochemical response after [^177^Lu]Lu-PSMA RLT [20, 28, 33, 55]. However, the results are inconsistent. Heck et al. [20] showed that visceral metastasis is independently associated with worse PSA response, clinical PFS (3.1 vs. 5.9 months) and OS (7.6 vs. 14.0 months). Also, in other studies, the multivariate analysis indicated the presence of visceral metastasis as an independent predictor of shorter OS [33, 35, 57]. Rahbar et al. [28] reported that the presence of visceral metastases independently lowers the biochemical response (odds ratio: 3.732 [95% CI, 1.412–9.864]). Also, Barber et al. [34] reported that the presence of visceral metastasis is associated with shorter imaging-based PFS (univariate analysis: HR: 1.90 [95% CI, 1.28–2.84], *P* = 0.002; multivariate analysis: *P* = 0.08) and OS (multivariate analysis: HR: 1.69 [95% CI, 1.02–2.80]). Furthermore, in the recent multicenter study, Ahmadzadehfar et al. [4] showed that liver metastasis worsens OS (HR: 2.394 [95% CI, 1.818–3.153]) while lung metastasis does not influence the survival [4]. However, they did not report the value of the presence of visceral (lung plus liver and other organs) metastases in cumulation. Also, the absence of visceral metastasis predicted longer PSA PFS (HR: 0.51) [37]. Contrarily, Ferdinandus et al. [24] and Derlin et al. [38] reported that liver metastasis does not influence the PSA response. Additionally, no correlation was demonstrated between visceral metastasis and PSA response [55] or OS [23, 30, 39], in other studies. However, Rahbar et al. [23] claimed that the presence of visceral metastasis showed poorer OS but did not reach the statistical significance.

The presence of visceral metastasis per se indicates an aggressive disease and shortens survival [47, 58]. Hence, there is a higher probability that these patients fail to respond to standard treatments and receive [^177^Lu]Lu-PSMA RLT. Additionally, the ratio of patients with visceral metastasis is relatively high and variable in the studies, which may have a negative impact on the statistical analysis justifying the absence of correlation between visceral metastasis and prognosis.

### Bone metastasis

Bone metastasis is a common manifestation in prostate cancer [59, 60] shortening overall survival [61]. It is the most common site of metastasis in prostate cancer [62]. Higher numbers of bone metastases indicate a high-risk disease [47]. However, the results regarding the impact of the presence of bone metastasis in response to [^177^Lu]Lu-PSMA RLT are contradictory. In a large multicenter study, the presence of bone metastasis predicted worse OS (HR: 3.703 [95% CI, 1.900–7.214]; *P* < 0.0001) in the multivariate analysis [4]. Also, Barber et al. [34, 63] showed that the presence of bone metastasis predicts shorter OS (HR: 5.90 [95% CI, 2.15–16.19]) and imaging-based PFS (HR: 1.84 [95% CI, 1.10–3.07]) only in the univariate analysis. In another multicenter study, the absence of bone metastasis was predictive of longer PSA PFS [37]. Furthermore, the number of bone metastasis was predictive of shorter OS in the univariate analysis in one study [35]. Nevertheless, the presence [28, 55] or number [24, 26] of bone metastasis did not impact the biochemical response to [^177^Lu]Lu-PSMA RLT nor did change OS [23, 39], in other surveys. It may attribute to the fact that patients receiving [^177^Lu]Lu-PSMA RLT are metastatic CRPC patients in an advanced state and most of them already have bone metastases, in almost all studies [4, 23, 24, 28, 55]. Hence, the low number of patients without bone involvement in these studies may cause bias in the statistical analysis. Additionally, studies have not separately investigated those patients with bone-only metastasis since concomitant metastasis in other organs may have an impact on the outcome. All in all, most of the eligible patients for [^177^Lu]Lu-PSMA RLT have bone metastases; therefore, it would not impact the decision-making. However, the influence of the bone-only metastases and the extent of bone involvement on survival require further clarification.

### Lymph node metastasis

The patients with advanced mCRPC rarely present with lymph node-only metastasis [58]. They have a better outcome than those with metastasis in other organs [58]. Multiple studies have reported that the presence of lymph node metastasis does not impact the outcome (including PSA response or survival) after [^177^Lu]Lu-PSMA RLT [4, 24, 28, 30, 38, 39, 50, 55]. Noteworthy, the studies rarely have evaluated the patients with lymph node–only metastasis [50]. Recently, Gafita et al. [33] reported that patients having concomitant bone and lymph node metastases have poorer OS following [^177^Lu]Lu-PSMA RLT compared to those with only lymph node metastases (HR: 1.39). Also, they revealed that mCRPC patients with no distant lymph node metastasis (M1a) show longer PSA PFS (HR: 0.66) [37]. Patients receiving [^177^Lu]Lu-PSMA RLT are in the later stages of their disease and mostly have concomitant metastases in other organs.

### Alkaline phosphatase

Abnormal serum alkaline phosphatase (ALP) is one of the prognosticators of poor outcome in prostate cancer [47, 64]. In this regard, studies have reported that patients with abnormal pre-treatment ALP (≥ 220 U/L) have a worse biochemical response to [^177^Lu]Lu-PSMA RLT [28] and shorter OS [23, 30, 63]. Moreover, in multiple studies, rising ALP [20], abnormal level of ALP (>140, >220 [35] and > 240 U/L [26]), or higher pre-treatment levels [31, 39] were associated with lower survival mostly only on the univariate analysis. Bräuer et al. [30] depicted that the only prognostic factor for longer PSA PFS was normal pre-treatment ALP level. Likewise, Barber et al. [63] reported that the abnormal pre-treatment ALP (≥ 220 U/L) is the strongest predictor of worse OS (HR: 1.8 [95% CI, 1.08–3.10]) and shorter imaging-based PFS (HR: 2.13 [65% CI, 1.35–3.37]) in the multivariate analysis, in mCRPC patients regardless of previous chemotherapy with taxane.

Also, pre-treatment ALP level < 120 U/L and any change after the first cycle have been addressed in a study by Ahmadzadehfar et al. [32], which predicted longer survival. Additionally, Yordanova et al. [32] evaluated the Bone-specific Alkaline Phosphatase (BAP) and showed that the decreasing BAP after the treatment is predictive of longer OS. Nevertheless, Grubmüller et al. and Gafita et al. did not find a correlation between ALP and outcome following [^177^Lu]Lu-PSMA RLT [33, 36]. The patient population and the ratio of patients with distant metastases were similar to the other studies; however, they did not refer to the binary values (normal vs. abnormal) for analysis, in these two studies.

Overall, the increase in ALP levels suggests dysregulation in bone formation caused by metastases [65]; hence, abnormal ALP may indicate a higher burden of the disease with a poor prognosis. It seems that the abnormal level of ALP majorly impacts the survival, and it is a negative prognosticator of outcome following [^177^Lu]Lu-PSMA RLT.

### Lactate dehydrogenase

Lactate dehydrogenase (LDH) is considered a poor prognostic tumor marker in prostate cancer [66–68]. The prognostic value of LDH following [^177^Lu]Lu-PSMA RLT has been reported in some studies [31]. Heck et al. [20] demonstrated that the rising LDH levels independently predict worse clinical PFS and OS. LDH ≥ 225 mg/L was prognostic of shorter OS in a study by Ahmadzadehfar et al., in the univariate analysis [35]. Also, the same research group reported that a baseline LDH level of < 248 mg/L and any change after the first cycle could predict better survival [32]. However, others did not find a correlation between higher LDH and response to therapy or OS [24, 38, 39, 63]. Moreover, some authors showed that abnormal pre-treatment LDH has no prognostic value for predicting OS [23, 33, 36]. The results regarding LDH are controversial warranting further evaluations. Also, it is of importance to report the correlation using a unified endpoint, such as a normal cut-off value for future studies, as well.

### Bone marrow status

Poor bone marrow function is an important determinant of exclusion of patients from [^177^Lu]Lu-PSMA RLT. Patients should have acceptable parameters [69]. Lower levels of blood parameters may be caused by significant bone marrow infiltration with tumoral cells, prior toxic chemotherapies or radiation. Lower levels of hemoglobin (Hb) indicate poorer prognosis in mCRPC [68]. Barber et al. [63] showed that in mCRPC patients who underwent [^177^Lu]Lu-PSMA RLT, the low level of pre-treatment Hb (<7.5 mmol/L [12.1 g/dl]) strongly and independently predicts worse OS in both groups of patients with a history of previous chemotherapy with taxane and taxane-naïve patients. Ahmadzadehfar et al. [35] and Gadot et al. reported the low Hb level (<10.4 and < 9.2 g/dl, respectively) was a strong predictor of poor OS, in the multivariate analysis. In another multicenter survey including 267 patients by Gafita et al. [37], lower Hb level was predictive of shorter OS (HR: 1.53) and higher Hb level was correlated with longer PSA PFS (HR: 0.32).

Interestingly, Ferdinandus et al. [24] reported that the higher level of pre-treatment platelet level is the most significant predictor of poor response to therapy. The negative impact of thrombocytosis has been documented in other malignancies, as well [24]. It has been documented that some cytokines induce thrombocytosis [24]. Also, circulating tumoral cells use thrombocytes to protect themselves from the immune system [24] advocating the negative impact of high platelet levels.

Contrarily, the lower levels of Hb [24, 36, 38, 50], white blood cells [24, 38, 39, 50], or platelet [38, 39] were not predictive factors in other studies. It, in part, owes to the pre-treatment screening and excluding those with poor bone marrow reserve.

Altogether, it can be inferred that patients with a low level of Hb have a poor prognosis and may show shorter survival after [^177^Lu]Lu-PSMA RLT. Otherwise, white blood cells or platelet do not seem to impact the outcome.

### Other serum markers

Factors mirroring renal and liver functions have been investigated to find a correlation with response to therapy. The higher level of gamma-glutamyl transferase (GGT), as a liver function test, showed a correlation in the univariate analysis, which was not confirmed on the multivariate assessment [24, 35]. Also, the abnormal level of C-reactive protein (CRP) was predictive for outcome in the univariate analysis [24, 35, 36].

The level of albumin [24, 39] and bilirubin was not predictive [24]. Additionally, aspartate aminotransferase (AST) and alanine transaminase (ALT) have shown no predictive value for the outcome [24, 30, 38]. However, in another study, lower albumin and higher AST levels were significant predictors of lower survival [35]. The hypothesis encouraging authors to investigate whether liver function tests predict survival might be related to the advanced disease status or liver metastases. However, these factors are assessed before the initiation of the therapy and patients with significant disturbed liver function do not meet the criteria to receive [^177^Lu]Lu-PSMA RLT. Hence, the minimally disturbed liver function test, which is not clinically significant, may not alter the outcome after [^177^Lu]Lu-PSMA RLT.

Moreover, Yordanova et al. [32] evaluated the prognostic value of neuroendocrine tumor markers. The presence of neuroendocrine features is proposed as a poor prognostic factor in PCa [32]. They showed that the decrease in pro-Gastrin-Releasing-Peptide and Chromogranin A levels after therapy predicts longer OS; however, the pre-treatment pro-Gastrin-Releasing-Peptide, Chromogranin A, and the change in the Chromogranin A level were not prognostic [32].

The results evaluating the liver function test is inconsistent. Also, the other biochemical parameters are sparsely evaluated in different surveys. Far more studies are required to detect the most predictive factors. Recent data proposed that other factors such as aggressive types of PC showing neuroendocrine differentiation or possessing somatic genomic alterations or specific germline mutations (BRCA2) may negatively influence the outcome, warranting future investigations [70].

### Age

The age of patients has been evaluated as a prognostic factor for [^177^Lu]Lu-PSMA RLT. Gadot et al. proposed older age as an independent predictor of longer OS after [^177^Lu]Lu-PSMA [39]. Heck et al. [20] reported that younger patients have poorer clinical PFS and OS. However, the association with OS was not confirmed on the multivariate analysis [20]. Likewise, Ferdinandus et al. [24] showed that the response to treatment in younger patients is poorer in only univariate analysis. The correlation between age and outcome after [^177^Lu]Lu-PSMA RLT was not confirmed in other studies [4, 23, 26, 40, 63]. The results are controversial regarding the age of the patients. Even though younger patients may reveal poorer response to [^177^Lu]Lu-PSMA RLT, the impact should be confirmed in further trials and age by itself should not alter the decision of commencing or cessation of the treatment.

### Performance status

The performance status of the patient is of importance to select patients for [^177^Lu]Lu-PSMA RLT. Commonly, those with acceptable performance status are eligible to receive this therapy [69]. It is hypothesized that patients with poor performance are those with a more advanced and poor prognostic disease. It seems that the lower Eastern Cooperative Oncology Group (ECOG) score correlates with a better outcome [4, 33, 35]. In line with that, in a multicenter survey, the authors [4] confirmed that patients with ECOG 0 and 1 reveal significantly longer OS compared to those with score 2 (16.9 vs. 9.6 vs. 6.3, respectively). Likewise, a multivariate analysis [26] showed that patients with ECOG ≤ 2 have longer OS compared to those with a score of > 2. However, it was not correlated with PSA response [26]. Moreover, Karnofsky score ≤ 80% was independently correlated with shorter OS (HR: 1.83 [95% CI, 1.07–3.14]) [63]. On the other hand, no correlation was depicted between ECOG and response to therapy or OS in other studies [24, 39, 40]. In one study by Yadav et al. [26], visual analgesic score, analgesic score, and Karnofsky Performance Status scale were not predictive of PSA response. It is of note that the endpoints were different in these studies (OS vs. PSA response), as well as the performance status of the patients. Patients with ECOG 0–1 may be in earlier stages of the disease; therefore, they may show a better outcome. Further studies are demanded to confirm the results.

### Analgesic intake

Bone pain is frequently seen in mCRPC patients, which may subside by [^177^Lu]Lu-PSMA RLT [27]. Ferdinandus et al. [24] showed that regular need for analgesic medication is strongly associated with poor response to [^177^Lu]Lu-PSMA RLT. Also, in two separate studies, Ahmadzadehfar et al. reported the inverse impact of regular need for analgesia or opioids with OS [35] and PSA response [40]. Patients with more advanced disease or higher disease burden may need more pain medication; therefore, the regular need for analgesia per se indicates more advanced disease.

### Gleason score

A higher Gleason score indicates a higher risk of metastases [47]. Hence, several studies have tried to find its prognostic value. Ferdinandus et al. [24] revealed that a Gleason score of 10 had a negative impact on any PSA decline following [^177^Lu]Lu-PSMA RLT in the univariate analysis; however, this did not keep its significance in the multivariate analysis. Likewise, Gadot et al.’s results [39] showed a lack of association between Gleason score and a PSA decline of 20% or more following [^177^Lu]Lu-PSMA RLT. The findings were in line with the lack of association between Gleason score and either any PSA decline or ≥ 50% PSA decline following [^177^Lu]Lu-PSMA RLT, reported by Yadav et al. [26]. Moreover, no significant association was identified between Gleason score [40, 50] or Gleason grade [38] and PSA response to [^177^Lu]Lu-PSMA RLT. Heck et al. [20] did not recognize the Gleason score (8–10 vs. 6–7) as a predictive factor of either maximum PSA decline of ≥50%, clinical PFS or OS. Furthermore, Gafita et al. [33] did not identify a Gleason score of 8 or more as a predictor of OS or imaging-based PFS, which was similar to findings of Suman et al.’s study [48], delineating Gleason score not to be predictive of either PFS or OS. It seems that the Gleason score has no significant impact on response rate or patients’ survival following [^177^Lu]Lu-PSMA RLT.

## Conclusion

**Fig. 1:**
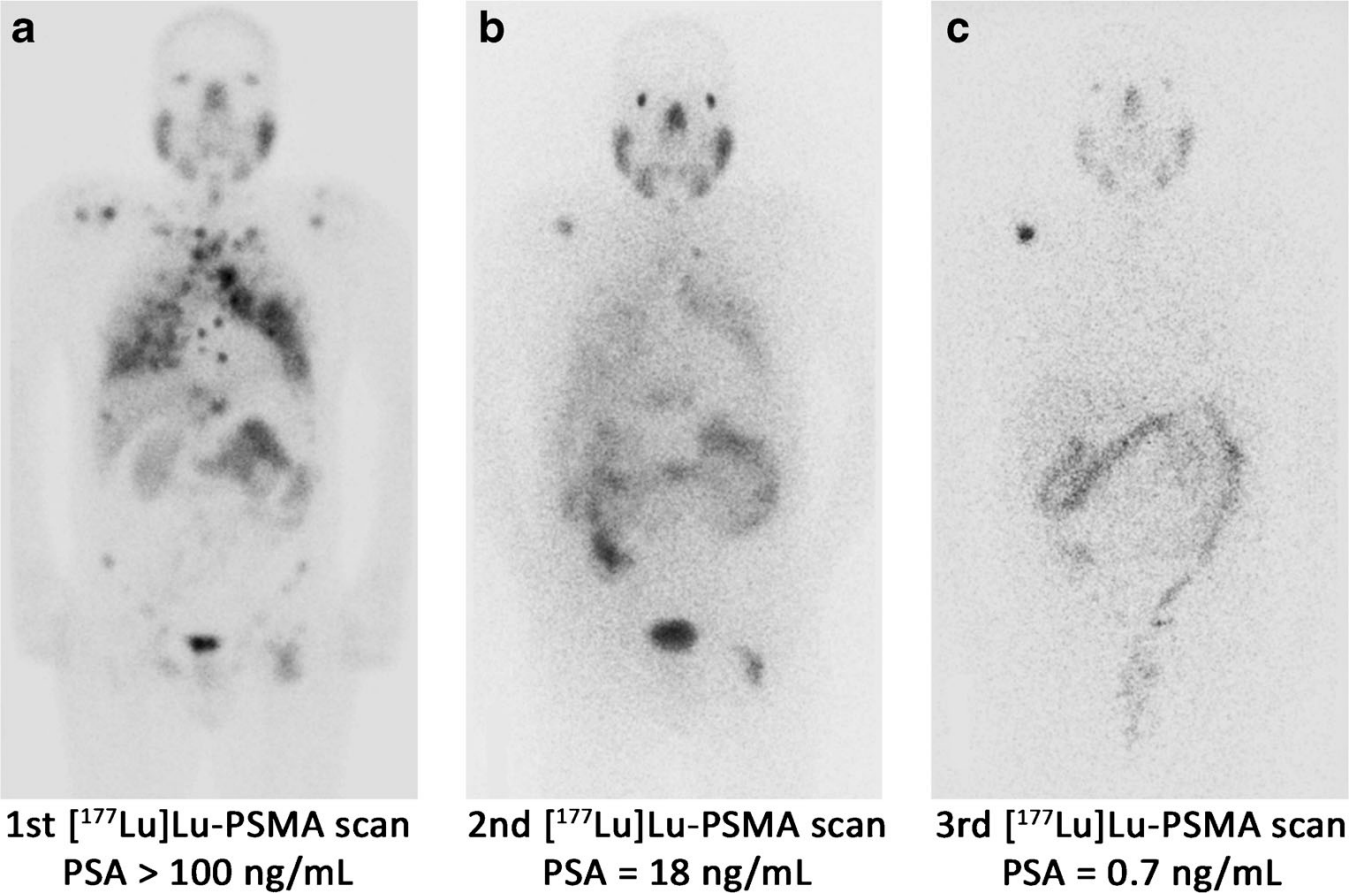
A 56-year-old male with metastatic castration-resistant prostate cancer involving the cervical, mediastinal, and abdominopelvic lymph nodes, multiple bones, and bilateral lungs underwent 3 cycles of Lutetium-177-prostate-specific membrane antigen ([^177^Lu]Lu-PSMA) radionuclide therapy. The sequential post-treatment (PT) planar whole-body [^177^Lu]Lu-PSMA images (**a**–**c**) and pre-therapy prostate-specific antigen (PSA) levels indicate a favorable response to treatment

**Fig. 2:**
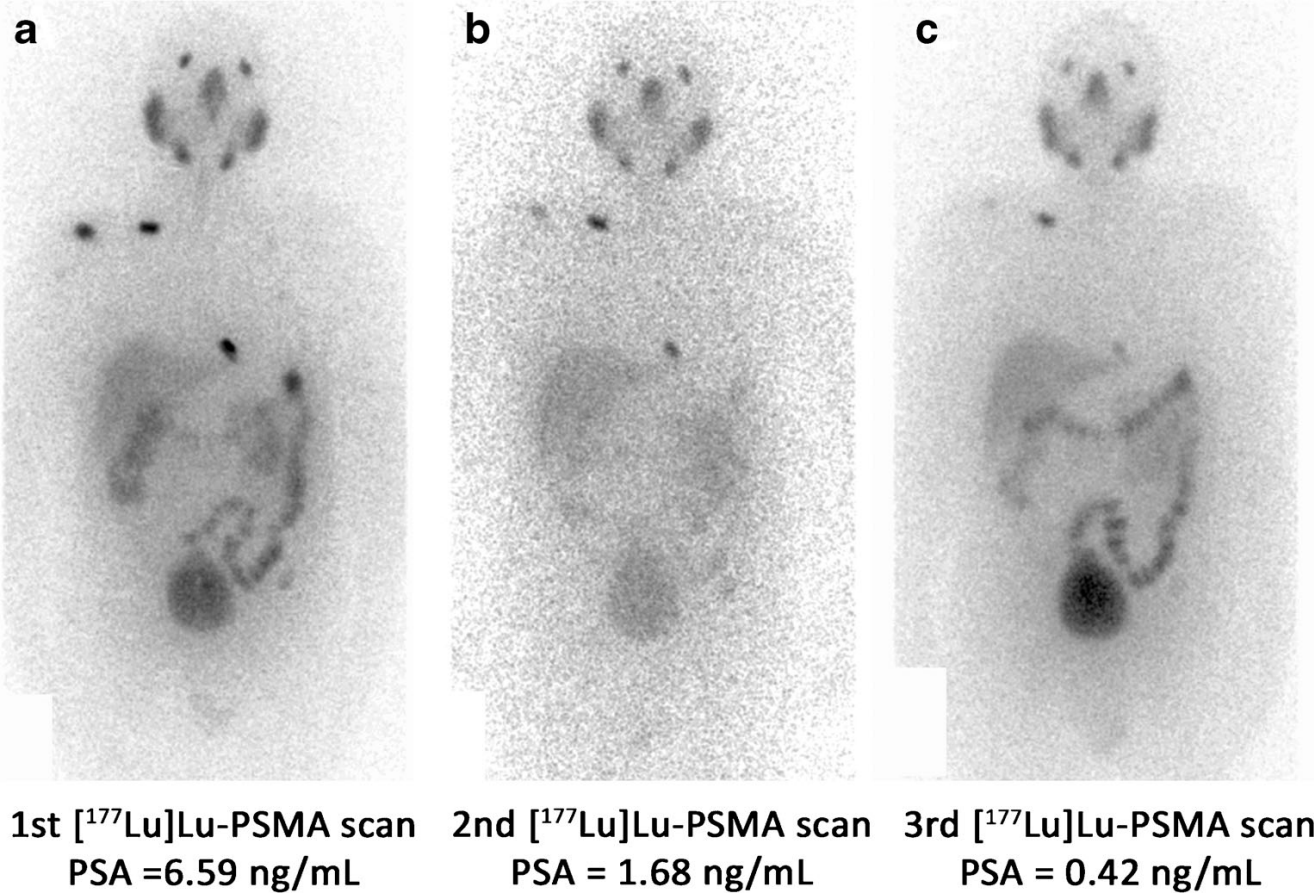
A 75-year-old male with metastatic castration-resistant prostate cancer underwent 3 cycles of Lutetium-177-prostate-specific membrane antigen ([^177^Lu]Lu-PSMA) radionuclide therapy. He had multiple bone metastases in the upper cervical vertebra, right humerus, right clavicle, both scapulae, bilateral ribs, left iliac bone, spinous process of the mid lumbar spine. The first post-treatment (PT) planar whole-body [^177^Lu]Lu-PSMA image (**a**) showed PSMA-avid lesions in the upper cervical vertebra, right humerus, right clavicle, a left lower rib, as well as the spinous process of the mid lumbar spine (seen in the posterior view). The subsequent PT images (**b** and **c**) demonstrated decreasing uptake in the lesions, and the prostate-specific antigen (PSA) level was declining. The PSA level was stable after 2 months of the third cycle (0.45 ng/mL). The response was favorable despite uptake in a few lesions in the post-therapy image

Radioligand therapy with [^177^Lu]Lu-PSMA is demonstrating striking results in heavily treated mCRPC patients who have exhausted all standard treatments. The sequential images of two mCRPC patients are illustrated in Figs. 1 and 2. The remarkable response rate and survival benefits in the primary studies made [^177^Lu]Lu-PSMA RLT a widely accepted option, in countries where it is available, while the phase III clinical trial is still ongoing. Patients with PSMA-avid lesions are selected for therapy. However, the response and survival range are wide in most studies. Approximately, 20–30% of patients do not respond to [^177^Lu]Lu-PSMA RLT indicating that there must be some other underlying mechanisms other than PSMA avidity. Several studies have investigated a multitude of factors to detect those with prognostic value. Among all parameters, any PSA decline after the first cycle of the therapy is recognized as the most robust predictive factor of prolonged survival. Also, future studies may confirm the prognostic significance of PSA response after the conclusion of the treatment, the presence of visceral metastasis and abnormal pre-treatment ALP. On the other hand, factors, such as Gleason score, primary PSA level, and PSA doubling time, have no impact on the response to [^177^Lu]Lu-PSMA RLT. Some other factors, including age, liver function test, and prior therapies, except for chemotherapy, possibly do not remarkably influence the outcome. Additionally, more investigations are needed to precisely define the prognostic value of other parameters, namely pre-treatment levels of Hb and LDH, performance status, analgesic intake, bone or lymph node metastases, the intensity of uptake in the pre-treatment PET/CT, and pre-treatment PSA level, as well as the dose and number of cycles of the therapy.

Noteworthy, there were limitations to our review. First, this article was a narrative review and we intentionally did not conduct a systematic review at this stage, to avoid any misleading reports; because the [^177^Lu]Lu-PSMA RLT has been mainly performed in clinical practice in the last few years, and the authors believe that the current number of the publications may not reliably reflect the factors predicting treatment response. Moreover, there was not a unified treatment protocol throughout the studies. The number of administered cycles and the amount of administered activities were different. Moreover, an inhomogeneous patient population with a history of different prior treatments and performance status were included in the investigations. Additionally, studies have evaluated the response in different time points with inconsistent endpoints. Also, most of the studies were retrospective, and a significant subfraction of them was from Germany. Hence, the overlap of the patients’ population was plausible. Finally, some factors were evaluated by only a few researchers. Importantly, the value of early administration of [^177^Lu]Lu-PSMA RLT, which may show added survival benefits, was scarcely addressed. Therefore, future prospective randomized trials are required to detect the independent prognostic factors, and to further determine the clinical and survival benefits of [^177^Lu]Lu-PSMA RLT.
